# Supplementary material for: Targeted suppression of mTORC2 reduces seizures across models of epilepsy
Source: Nat Commun. 2023 Nov 14;14:7364. doi: 10.1038/s41467-023-42922-y (PMC10645975; doi:10.1038/s41467-023-42922-y)
Supplement: Supplementary file 1 — Supplementary Information [file 41467_2023_42922_MOESM1_ESM.pdf]

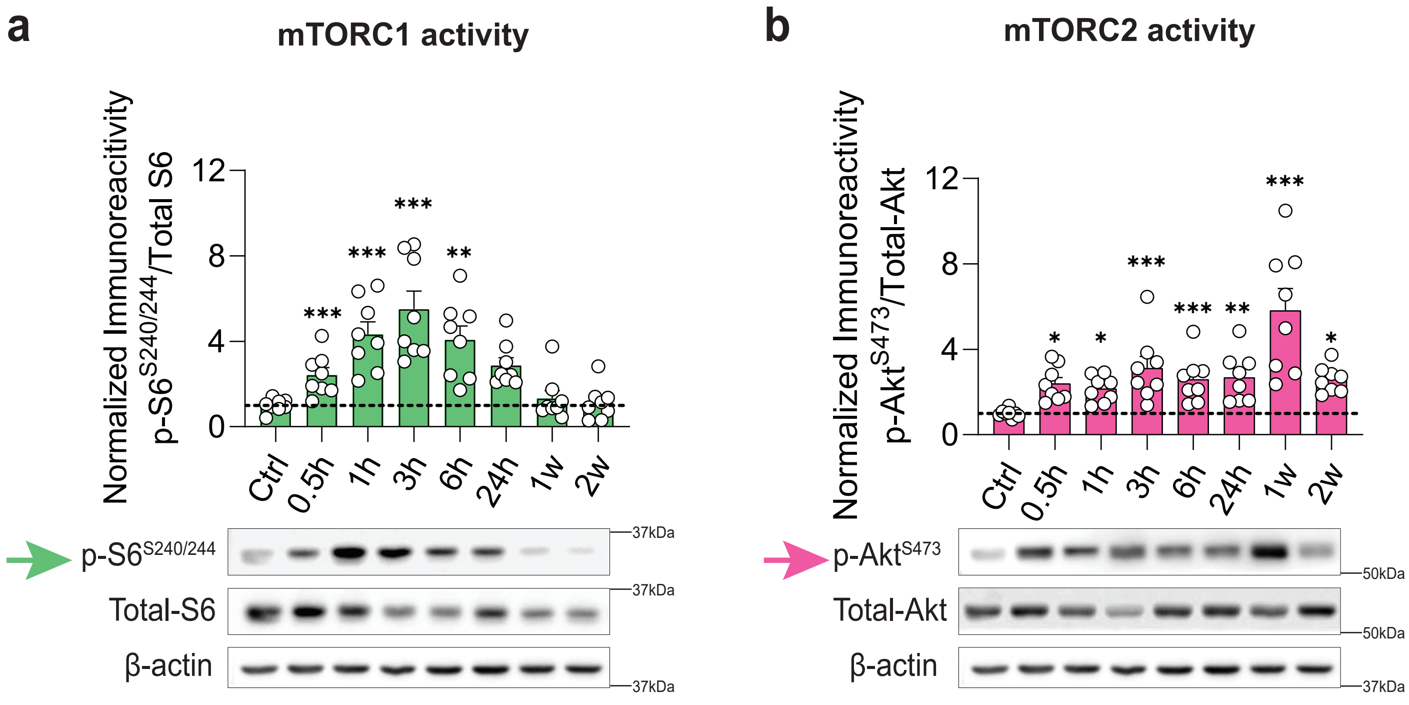

Supplementary Figure 1. KA activates mTORC2 more persistently than mTORC1.

a, b, mTOR complexes activity after KA injection. Quantification and representative western blots of mTORC1 activity measured by p-S6$^{S240/244}$ (**a,** F = 11.58, P<0.001) and mTORC2 activity measured by p-Akt^{S473} (**b,** F = 47.94, P<0.001) in the hippocampus of WT mice (n = 8 per group, each timepoint is compared to naive (ctrl) mice). Data are represented as means ± s.e.m. *P<0.05, **P<0.01, ***P<0.001.

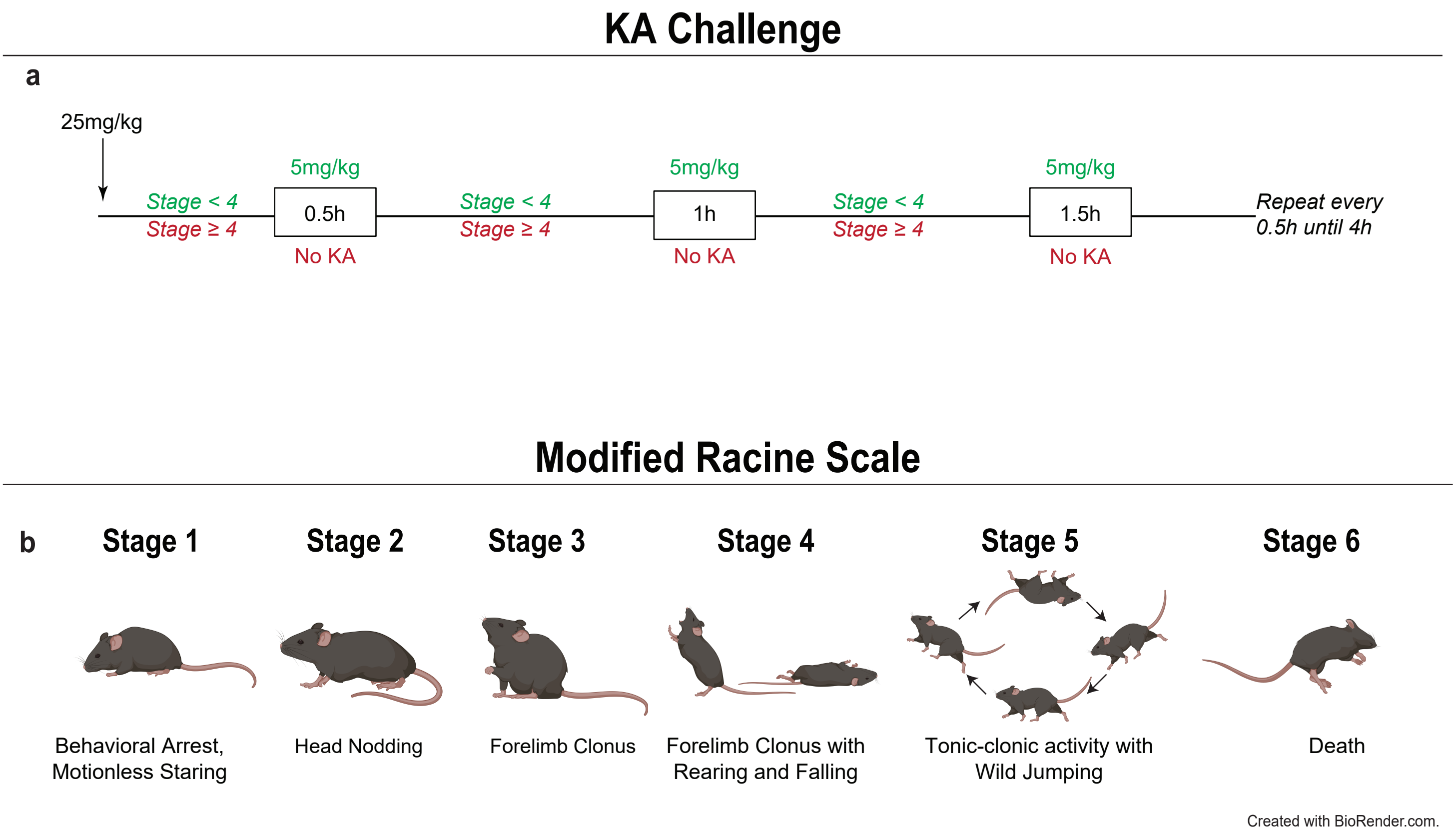

Supplementary Figure 2. KA-induced behavioral seizure scoring. a, b, Schematic for KA behavioral seizure challenge (**a**) and modified Racine Scale (**b**).

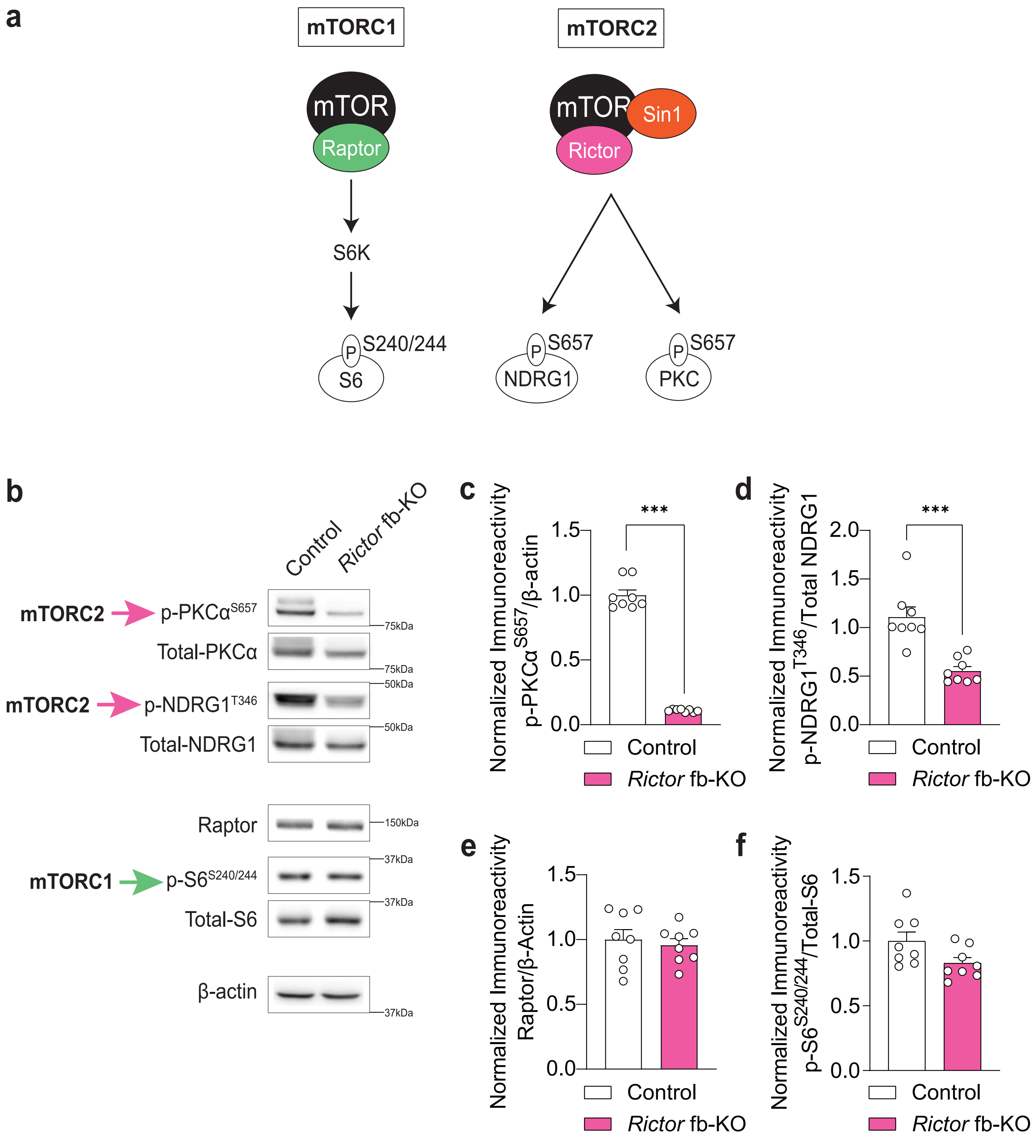

Supplementary Figure 3. Genetic deletion of *Rictor* selectively reduces mTORC2 activity. a-f, mTOR activity in *Rictor* fb-KO mice. **a,** Schematic of mTORC1 and mTORC2 targets. Representative western blots (**b**) and quantification of mTORC2 activity measured by p-PKCα^{657} (**c,** U = 0, P <0.001) and p-NDRG1^{T346} (**d,** U = 1, P <0.001) from the cortex of control and *Rictor* fb-KO mice. Quantification of Raptor levels (**e,** t = 0.4578, P = 0.65) and mTORC1 activity measured by p-S6$^{S240/244}$ (**f,** t = 2.092, P = 0.06) from the cortex of control and *Rictor* fb-KO mice (n = 8 per group). Data are represented as means ± s.e.m. *P<0.05, **P<0.01, ***P<0.001.

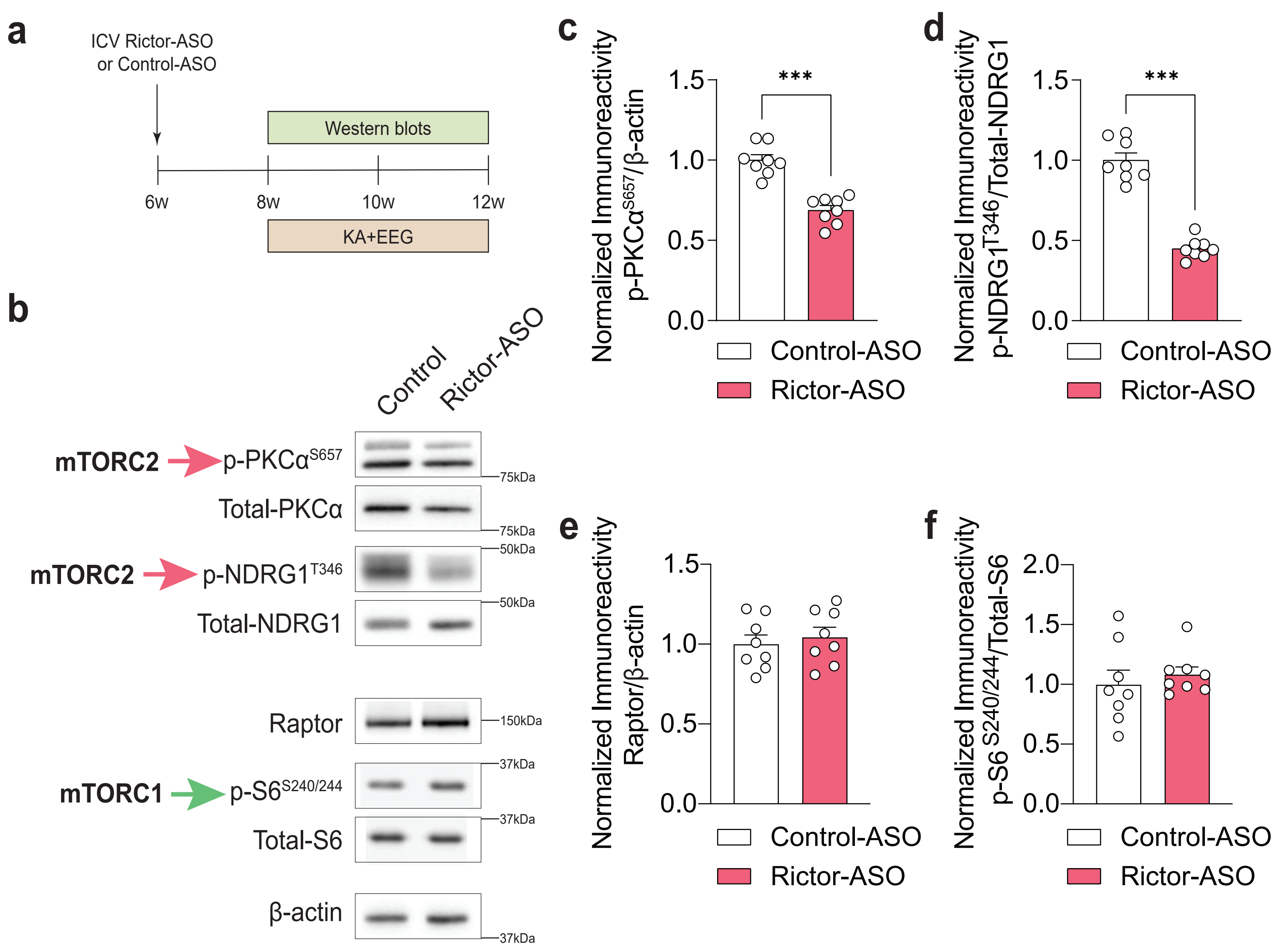

Supplementary Figure 4. ASO-mediated suppression of Rictor selectively reduces mTORC2 activity.

a, Timeline of ASO treatment, western blots and EEG recording from WT mice. Representative western blots (**b**) and quantification of mTORC2 activity measured by p-PKCα^{657} (**c,** t = 6.917, P <0.001) and p-NDRG1^{T346} (**d,** t = 10.94, P <0.001) from the cortex of control and Rictor-ASO treated mice. Quantification of Raptor levels (**e,** t = 0.6508, P = 0.526) and mTORC1 activity measured by p-S6S240/244 (**f,** t = 0.3661, P = 0.72) from the cortex of control and Rictor-ASO treated mice (n = 8 per group). Data are represented as means ± s.e.m. *P<0.05, **P<0.01, ***P<0.001.

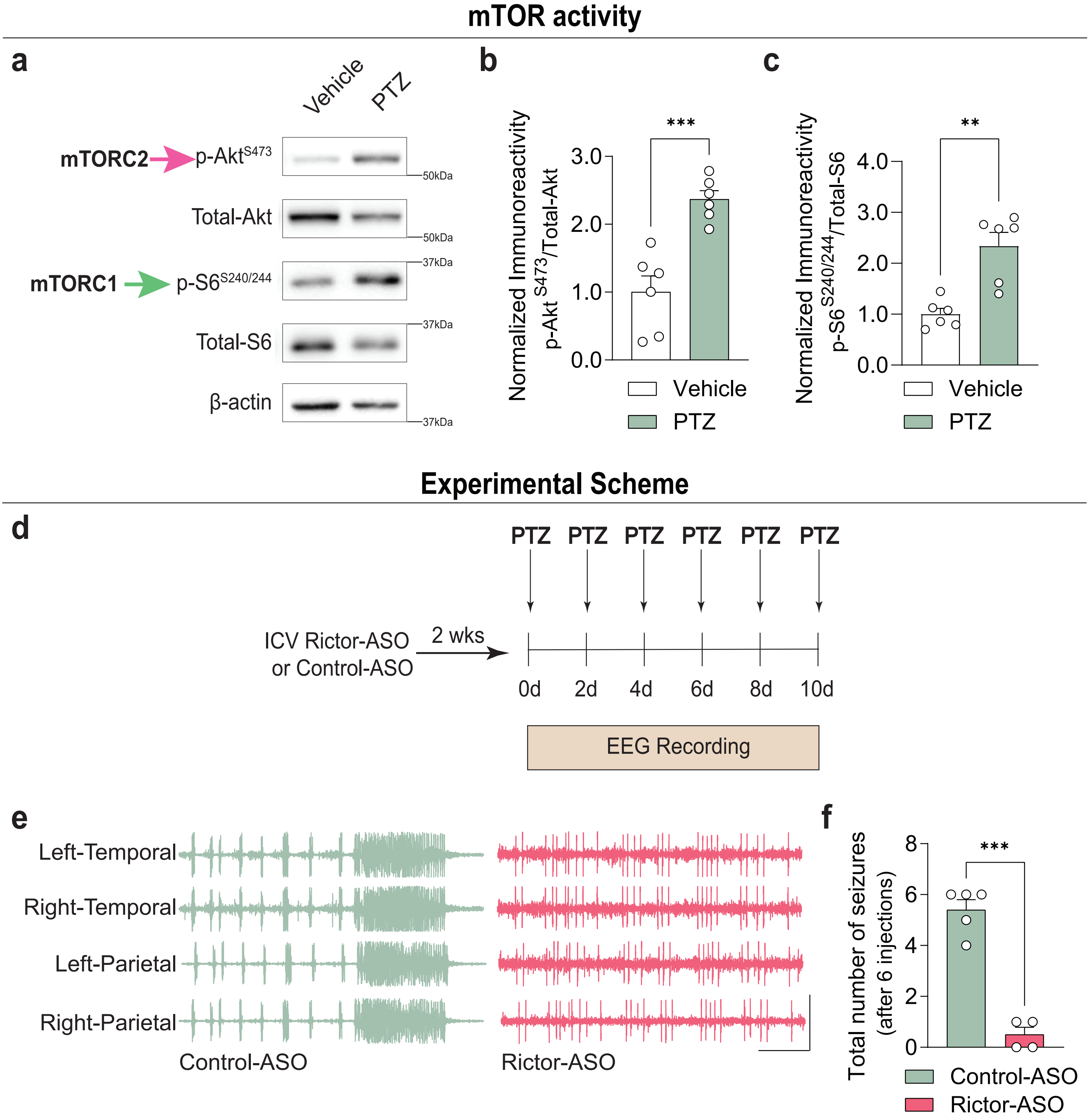

Supplementary Figure 5. Rictor-ASO treatment suppresses acute PTZ-induced seizures.

a-c, mTORC2 activity in the PTZ model. Representative western blots (a) and quantification of mTORC2 activity measured by p-Akt^{S473} (b, $t = 5.053$, $P < 0.001$) and mTORC1 activity measured by p-S6$^{S240/244}$ (**c,** $U = 1$, $P = 0.0043$) in the cortex of vehicle or PTZ-treated WT mice ($n = 6$ per group, mice were injected with 75 mg/kg PTZ or vehicle and tissue was collected 30 minutes after injection). d, Schematic for control-ASO or Rictor-ASO treatment in PTZ-kindling model. e-f, PTZ-induced seizures in control-ASO and Rictor-ASO treated mice. Representative EEG traces (e) and quantification (f) of total number of EEG seizures up to 1h post-PTZ injection in control-ASO ($n = 5$) and Rictor-ASO ($n = 4$) treated mice ($t = 9.933$, $P<0.001$). Data are represented as means ± s.e.m. *P<0.05, **P<0.01, ***P<0.001.

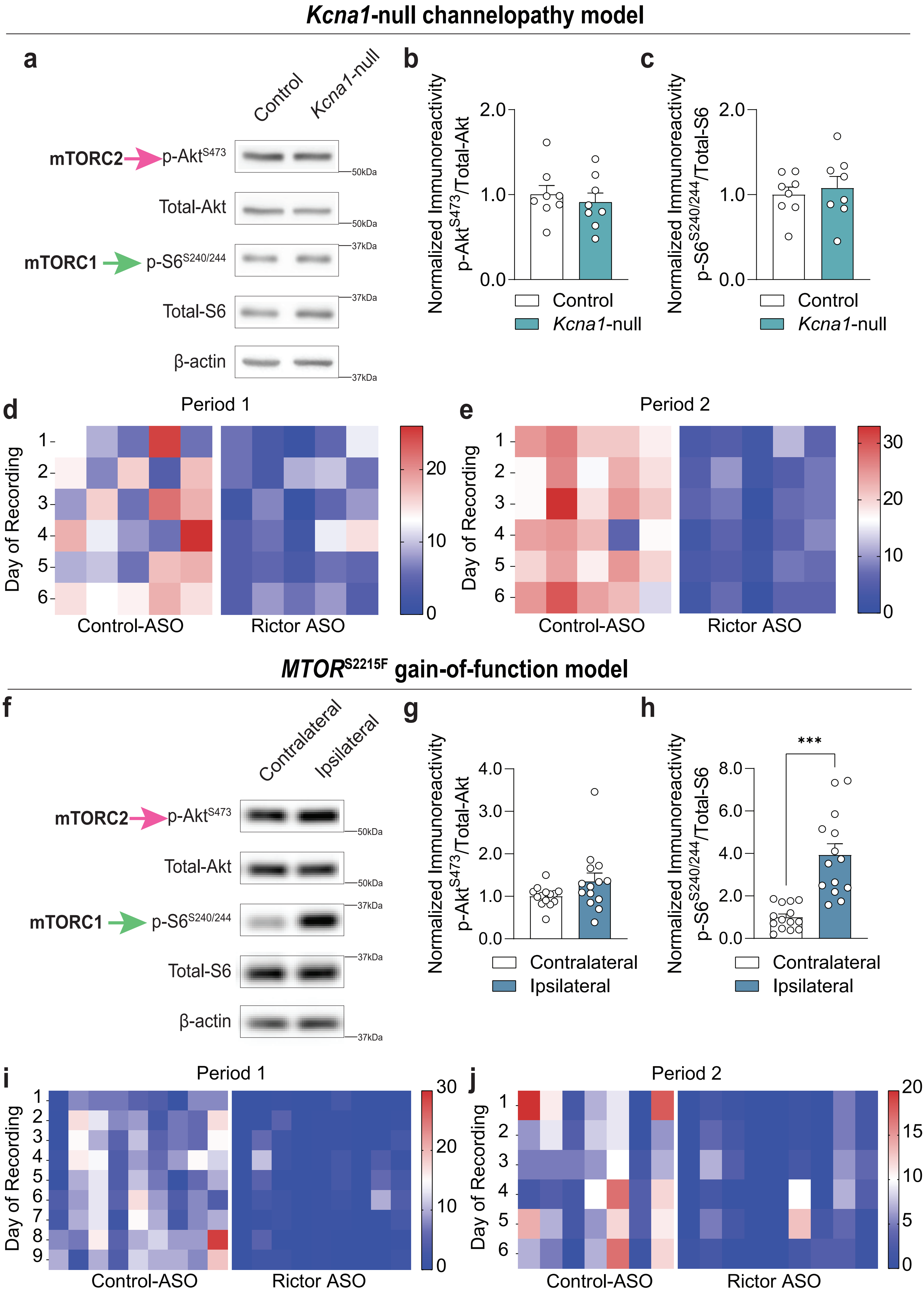

Kcna1-null channelopathy model
a
Control
Kcna1-null
mTORC2
p-Akt S473
Total-Akt
mTORC1
p-S6 S240/244
Total-S6
β-actin
50kDa
37kDa
b
Normalized Immunoreactivity
p-Akt S473/Total-Akt
2.0
1.0
0.0
Control
Kcna1-null
c
Normalized Immunoreactivity
p-S6 S240/244/Total-S6
d
Period 1
Day of Recording
Control-ASO
Rictor ASO
e
Period 2
MTOR S2215F gain-of-function model
f
Contralateral
Ipsilateral
g
4.0
3.0
h
***
8.0
6.0
i
j

Supplementary Figure 6. Rictor-ASO treatment suppresses seizures in two preclinical models of epilepsy. a-c, mTOR complex activity in 4-week-old *Kcna1*-null mice. Representative western blots (**a**) and quantification of mTORC1 (**b,** t = 0.1970, P = 0.85) and mTORC2 (**c,** t = 1.008, P = 0.3307) activity in control and *Kcna1*-null mice (n= 8 per group). **d-e**, Seizure frequency in Rictor-ASO treated *Kcna1*-null mice. Heatmap of number of seizures per day for Period 1 (**d,** P = 0.034) and Period 2 (**e,** P<0.001) in control and *Kcna1*-null mice (n = 5 per group). **f-h,** mTOR complex activity in 6-week-old *MTOR*S^{2215F} mice. Representative western blots (**f**) and quantification of mTORC1 (g, t = 5.392, P < 0.001) and mTORC2 (**h,** U = 61, p = 0.09) activity in *MTOR*^{S2215F} mice. **i-j,** Seizure frequency in Rictor-ASO treated *MTOR*^{S2215F} mice. Heatmap of number of seizures per day for Period 1 (**i,** P<0.001) and Period 2 (**j,**P<0.001) in Control (n = 9, 7) or MTORS2215F (n = 9, 9) mice. Data are represented as means ± s.e.m. *P<0.05, **P<0.01, ***P<0.001.

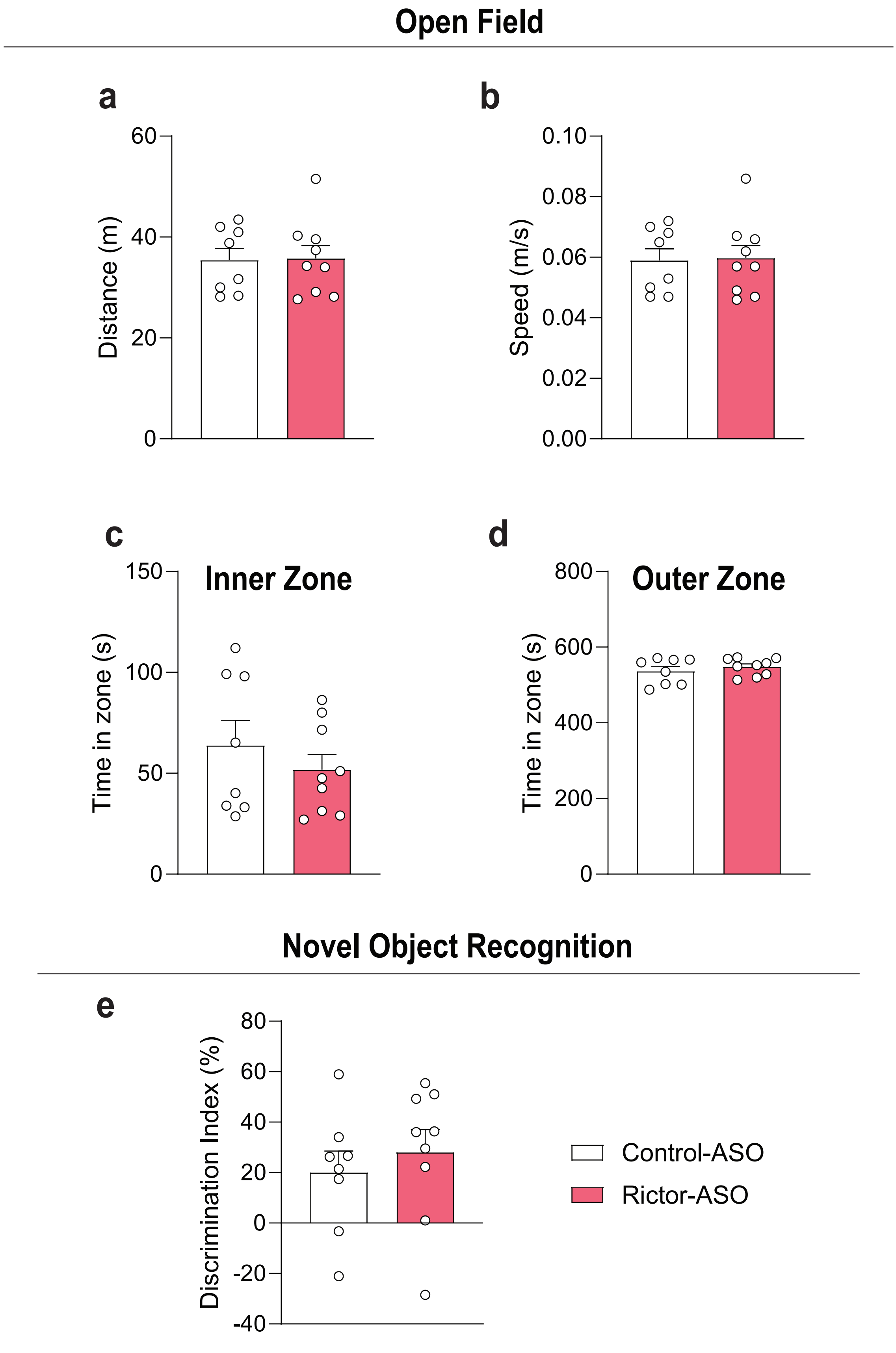

Supplementary Figure 7. Rictor-ASO treatment does not affect activity levels or learning and memory in wildtype mice. a-d, Activity levels in 8-week-old control or Rictor-ASO treated mice. Distance traveled (**a**, t = 0.101, P = 0.9209), average speed (**b,** t = 0.1165, P = 0.9088), time spent in inner zone (**c,** t = 0.8564, P = 0.4502) and time spent in outer zone (**d,** t = 0.8568, P = 0.4050) in the open field arena. Object recognition memory in control and Rictor-ASO treated mice as measured by discrimination index (**e,** t = 0.6425, P = 0.5302) in the novel object recognition test (n = 8 mice per group). Data are represented as means ± s.e.m. *P<0.05, **P<0.01, ***P<0.001.
