# Supplementary material for: Targeted suppression of mTORC2 reduces seizures across models of epilepsy
Source: Nat Commun. 2023 Nov 14;14:7364. doi: 10.1038/s41467-023-42922-y (PMC10645975; doi:10.1038/s41467-023-42922-y)
Supplement: Supplementary file 3 — Reporting Summary [file 41467_2023_42922_MOESM3_ESM.pdf]

nature portfolio

Corresponding author(s): Mauro Costa-Mattioli

Last updated by author(s): Oct 4, 2023

Reporting Summary

Nature Portfolio wishes to improve the reproducibility of the work that we publish. This form provides structure for consistency and transparency in reporting. For further information on Nature Portfolio policies, see our Editorial Policies and the Editorial Policy Checklist.

Statistics

For all statistical analyses, confirm that the following items are present in the figure legend, table legend, main text, or Methods section.

n/a	Confirmed	
☐	☒	The exact sample size (*n*) for each experimental group/condition, given as a discrete number and unit of measurement
☐	☒	A statement on whether measurements were taken from distinct samples or whether the same sample was measured repeatedly
☐	☒	The statistical test(s) used AND whether they are one- or two-sided<br>*Only common tests should be described solely by name; describe more complex techniques in the Methods section.*
☐	☒	A description of all covariates tested
☐	☒	A description of any assumptions or corrections, such as tests of normality and adjustment for multiple comparisons
☐	☒	A full description of the statistical parameters including central tendency (e.g. means) or other basic estimates (e.g. regression coefficient) AND variation (e.g. standard deviation) or associated estimates of uncertainty (e.g. confidence intervals)
☐	☒	For null hypothesis testing, the test statistic (e.g. F, t, r) with confidence intervals, effect sizes, degrees of freedom and P value noted<br>*Give P values as exact values whenever suitable.*
☒	☐	For Bayesian analysis, information on the choice of priors and Markov chain Monte Carlo settings
☒	☐	For hierarchical and complex designs, identification of the appropriate level for tests and full reporting of outcomes
☒	☐	Estimates of effect sizes (e.g. Cohen's d, Pearson's r), indicating how they were calculated

Our web collection on statistics for biologists contains articles on many of the points above.

Software and code

Policy information about availability of computer code

Data collection	LICOR Odyssey (Western Blotting), AZURE 600 (Western Blotting), Powerlabs 16/35 (EEG)
Data analysis	LabChart 8 (EEG), Image J v2.14.0 (Western Blotting) Graphpad Prism 9 (Analysis) , R studio (Phosphoproteomics)

For manuscripts utilizing custom algorithms or software that are central to the research but not yet described in published literature, software must be made available to editors and reviewers. We strongly encourage code deposition in a community repository (e.g. GitHub). See the Nature Portfolio guidelines for submitting code & software for further information.

Data

Policy information about availability of data

All manuscripts must include a data availability statement. This statement should provide the following information, where applicable:

- Accession codes, unique identifiers, or web links for publicly available datasets
- A description of any restrictions on data availability
- For clinical datasets or third party data, please ensure that the statement adheres to our policy

The data sets generated during and/or analyzed during the current study are available from the corresponding author upon reasonable request. Source data are provided with this paper. The mass spectrometry proteomics data have been deposited to the ProteomeXchange Consortium via the PRIDE partner repository with the dataset identifier PXD045878.

Human research participants

Policy information about studies involving human research participants and Sex and Gender in Research.

Reporting on sex and gender	N/A
Population characteristics	N/A
Recruitment	N/A
Ethics oversight	N/A

Note that full information on the approval of the study protocol must also be provided in the manuscript.

Field-specific reporting

Please select the one below that is the best fit for your research. If you are not sure, read the appropriate sections before making your selection.

[x] Life sciences [ ] Behavioural & social sciences [ ] Ecological, evolutionary & environmental sciences

For a reference copy of the document with all sections, see nature.com/documents/nr-reporting-summary-flat.pdf

Life sciences study design

All studies must disclose on these points even when the disclosure is negative.

Sample size	No statistical methods were used to pre-determine sample sizes, but our sample sizes are selected based on previous studies published in the field. (Chen et al., Nature Medicine, 2019; Ehninger et al., Nature Medicine, 2008, Sztainberg et al., Nature, 2015; Kaplan et al., PNAS, 2017, Shao et al., Science Translational Medicine, 2022, Lenk et al., Annals of Neurology, 2020; Patra et al., Epilepsia, 2019.)
Data exclusions	No data was excluded from the analysis
Replication	All attempts at replication were successful. Each experiment was replicated independently at least twice.
Randomization	For molecular, behavioral and electrophysiological studies, mice were randomly assigned to control and experimental groups (online methods)
Blinding	These experiments were performed and analyzed blind to treatment conditions and/or genotype, information which was unveiled postanalysis.

Reporting for specific materials, systems and methods

We require information from authors about some types of materials, experimental systems and methods used in many studies. Here, indicate whether each material, system or method listed is relevant to your study. If you are not sure if a list item applies to your research, read the appropriate section before selecting a response.

Materials & experimental systems

n/a	Involved in the study	
[ ]	[x]	Antibodies
[ ]	[x]	Eukaryotic cell lines
[x]	[ ]	Palaeontology and archaeology
[ ]	[x]	Animals and other organisms
[x]	[ ]	Clinical data
[x]	[ ]	Dual use research of concern

Methods

n/a	Involved in the study	
[x]	[ ]	ChIP-seq
[x]	[ ]	Flow cytometry
[x]	[ ]	MRI-based neuroimaging

Antibodies

Antibodies used	Primary Antibodies were purchased from Cell Signaling Technology (Danvers, MA): Raptor (#2280), Rictor (#2114), p-S6 (Ser240/244, #5364), p-Akt (Ser473, #9271), S6 (#2217), Akt (#9272), GFP (#2956), p-PKCalpha (S657, #9371), p-NDRG1 (T346, #3217), PKCalpha (#2056), Total-NDRG1 (#9408), HA (#3734), β-actin (#3700). Secondary antibodies were purchased from Proteintech (Rosemont, IL) Mouse (SA00001-1), Rabbit (SA00001-2).

Validation	All antibodies used in this study are commercially available and validated antibodies. Validation is visible at cellsignal.com for primary antibodies or ptglab.com for secondary antibodies.

Eukaryotic cell lines

Policy information about cell lines and Sex and Gender in Research

Cell line source(s)	ATCC CRL-11268 (Commercial)
Authentication	Cell lines were authenticated using STR profiling.
Mycoplasma contamination	Cell-lines tested negative for mycoplasma contamination via a luminescent assay for mycoplasma metobolites.
Commonly misidentified lines (See ICLAC register)	No commonly misidentified cell-lines were used in this study.

Animals and other research organisms

Policy information about studies involving animals; ARRIVE guidelines recommended for reporting animal research, and Sex and Gender in Research

Laboratory animals	All animals used in this study were of the species Mus musculus of the strain C57BL/6 or Swiss/CD1 Animals used for behavioral experiments were both males and females 6-15 weeks old (see Online methods). The Kcna1-null mice were on the C57BL/6 background.
Wild animals	No wild animals were used in this study.
Reporting on sex	Sex differences were not measured and male and female mice were used for each experiment because epilepsy does not show a strong sex bias (Fiest et al., Neurology, 2017)
Field-collected samples	No field collected samples were used in this study.
Ethics oversight	Animal care and experimental procedures were approved by the institutional animal care and use committee of Baylor College of Medicine, according to US National Institutes of Health Guidelines (Online Methods/mouse husbandry) and the French Ministry of Research (no. APAFIS#3506).

Note that full information on the approval of the study protocol must also be provided in the manuscript.
